# Exploring the Metabolism of (+)-[^18^F]Flubatine In Vitro and In Vivo: LC-MS/MS Aided Identification of Radiometabolites in a Clinical PET Study †

**DOI:** 10.3390/molecules23020464

**Published:** 2018-02-20

**Authors:** Friedrich-Alexander Ludwig, Steffen Fischer, René Smits, Winnie Deuther-Conrad, Alexander Hoepping, Solveig Tiepolt, Marianne Patt, Osama Sabri, Peter Brust

**Affiliations:** 1 Helmholtz-Zentrum Dresden-Rossendorf, Research Site Leipzig, Institute of Radiopharmaceutical Cancer Research, Permoserstraße 15, 04318 Leipzig, Germany; s.fischer@hzdr.de (S.F.); w.deuther-conrad@hzdr.de (W.D.-C.); p.brust@hzdr.de (P.B.); 2 ABX advanced biochemical compounds GmbH, Heinrich-Gläser-Straße 10-14, 01454 Radeberg, Germany; smits@abx.de (R.S.); hoepping@abx.de (A.H.); 3 Department of Nuclear Medicine, University Hospital Leipzig, Liebigstraße 18, 04103 Leipzig, Germany; solveig.tiepolt@medizin.uni-leipzig.de (S.T.); marianne.patt@medizin.uni-leipzig.de (M.P.); osama.sabri@medizin.uni-leipzig.de (O.S.)

**Keywords:** [^18^F]flubatine, NCFHEB, [^18^F]FLBT, radiometabolites, glucuronides, liquid chromatography–tandem mass spectrometry (LC-MS/MS), liver microsomes, positron emission tomography (PET), nicotinic acetylcholine receptors (nAChRs)

## Abstract

Both (+)-[^18^F]flubatine and its enantiomer (−)-[^18^F]flubatine are radioligands for the neuroimaging of α4β2 nicotinic acetylcholine receptors (nAChRs) by positron emission tomography (PET). In a clinical study in patients with early Alzheimer’s disease, (+)-[^18^F]flubatine ((+)-[^18^F]**1**) was examined regarding its metabolic fate, in particular by identification of degradation products detected in plasma and urine. The investigations included an in vivo study of (+)-flubatine ((+)-**1**) in pigs and structural elucidation of formed metabolites by LC-MS/MS. Incubations of (+)-**1** and (+)-[^18^F]**1** with human liver microsomes were performed to generate in vitro metabolites, as well as radiometabolites, which enabled an assignment of their structures by comparison of LC-MS/MS and radio-HPLC data. Plasma and urine samples taken after administration of (+)-[^18^F]**1** in humans were examined by radio-HPLC and, on the basis of results obtained in vitro and in vivo, formed radiometabolites were identified. In pigs, (+)-**1** was monohydroxylated at different sites of the azabicyclic ring system of the molecule. Additionally, one intermediate metabolite underwent glucuronidation, as also demonstrated in vitro. In humans, a fraction of 95.9 ± 1.9% (*n* = 10) of unchanged tracer remained in plasma, 30 min after injection. However, despite the low metabolic degradation, both radiometabolites formed in humans could be characterized as (i) a product of *C*-hydroxylation at the azabicyclic ring system, and (ii) a glucuronide conjugate of the precedingly-formed *N*8-hydroxylated (+)-[^18^F]**1**.

## 1. Introduction

**Figure 1:**
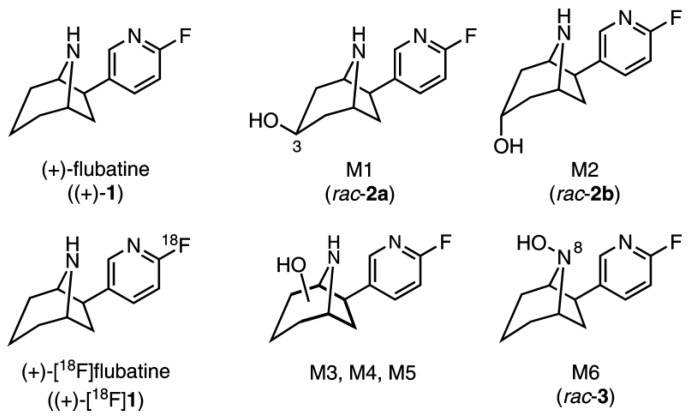
Chemical structures of (+)-flubatine ((+)-**1**), (+)-[^18^F]flubatine ((+)-[^18^F]**1**) and metabolites M1-M6, known from literature [4]. For M1, M2, and M6 the numbers in brackets refer to synthesized references (*rac*-**2a**, *rac*-**2b**, *rac*-**3**) used in this publication. References *rac*-**2a** and *rac*-**2b** have been described previously in [4].

Both enantiomers (+)-[^18^F]flubatine ((+)-(1*S*,5*R*,6*R*)-6-(6-[^18^F]fluoro-pyridine-3-yl)-8-azabicyclo- [3.2.1]octane, (+)-[^18^F]**1**, Figure 1) and (−)-[^18^F]flubatine ((−)-[^18^F]**1**) are radioligands for imaging of α4β2 nicotinic acetylcholine receptors (nAChRs) in brain by positron emission tomography (PET). Their development starting from the alkaloid (−)-epibatidine [1], originally isolated from the poison dart frog *Epipedobates anthonyi*, has been widely reported [2,3,4]. In the framework of preclinical and clinical studies, the metabolism of (−)-[^18^F]**1** in pigs [5], rhesus monkeys [6,7,8], and in humans [3,9,10,11] was examined mainly with the purpose of determination of the fraction of unchanged tracer over time to enable a metabolite correction of the PET data obtained. In human, (−)-[^18^F]**1** showed a high metabolic stability [9]. 

For the determination of the fraction of unchanged tracer, samples of arterial blood are collected, prepared, and analyzed by high performance liquid chromatography followed by online radioactive detection (radio-HPLC). Alternatively, eluate fractions from HPLC separation can be collected and inspected by a gamma counter. By radio-HPLC, in addition to the unchanged tracer, metabolites, that still bear the radioactive nuclide, named radiometabolites, can also be detected. However, due to the low concentrations, characterization of fluorine-18 bearing radiometabolites regarding their chemical structures is usually not possible in a direct manner. For structural elucidation additional investigations have to be conducted, e.g., by using the non-labelled parent compound of the tracer and liquid chromatrography-mass spectrometry (LC-MS) or, more selective, liquid chromatrography-tandem mass spectrometry (LC-MS/MS), instead of radio-HPLC [12]. For studying the metabolism in vivo, the non-labelled parent compound can be administered to small animals, e.g., rodents, in appropriate dosage to obtain samples of tissue or body liquids for examination by LC-MS and LC-MS/MS [13]. In addition to the fact that species differences have to be considered [14,15], in particular when making conclusions on the metabolism in humans, this approach has the advantage of covering all possible metabolic transformations, still summarized as phase I and phase II metabolism, but more exactly termed as ‘functionalization’ and ‘conjugation’ [16]. Drawbacks are, for instance, a possible ion suppression in MS detection caused by residual biological matrix [17]. Furthermore, animal studies have to be approved by the responsible authorities. To circumvent some of these issues, different in vitro models for metabolism studies have been described [18,19]. For basic investigations, especially liver microsomes, which contain cytochrome P450 enzymes and different types of transferases [19], are a suitable means that is easy to use, and of minimal cost. Since microsomes are also available from humans, the problem of species differences can be avoided to some extent. 

Such in vitro investigations have already been performed for (+)-**1** and (−)-flubatine ((−)-**1**), using liver microsomes from humans (HLM) and mice (MLM), as published by our group [4]. Incubations in the presence of β-nicotinamide adenine dinucleotide 2’-phosphate reduced tetrasodium salt (NADPH) resulted in the formation of hydroxylation products, provided in Figure 1. Published results from in vivo investigations of (+)-[^18^F]**1** showed the formation of one metabolite, that was not characterized further, but assumed to possibly pass the blood-brain barrier (BBB) and, therefore, confound brain PET images [4,5]. 

Considering this presumption and the importance of knowledge about the metabolic fate of a new tracer [20], we aimed to support the evaluation of (+)-[^18^F]**1** and investigated its metabolic pathways during a first clinical PET study, which compared the status of α4β2 nAChRs in patients with Alzheimer’s disease (AD) and healthy controls [21]. As shown in the present publication, we studied the metabolism of (+)-**1** in pigs and elucidated the structures of metabolites. For the first time, we investigated both phase I and II metabolism and performed incubations of (+)-**1** and (+)-[^18^F]**1** with HLM under conditions for oxidation and glucuronide conjugation. Resulting LC-MS/MS and radio-HPLC data provided the opportunity to conclude about the identity of formed in vitro radiometabolites. On that basis, we finally characterized radiometabolites formed in human after administration of (+)-[^18^F]**1**.

## 2. Results

### 2.1. Radiosynthesis of (+)-[^18^F]**1**

(+)-[^18^F]**1** was synthesized by nucleophilic substitution [22] under GMP conditions for human application. The product was obtained with a radiochemical purity >98.0% and molar activity >1.0 × 10^6^ GBq/mmol as described for (−)-[^18^F]**1** [23,24].

### 2.2. Synthesis of rac-8-Hydroxy-flubatine (rac-**3**)

**Figure 2:**
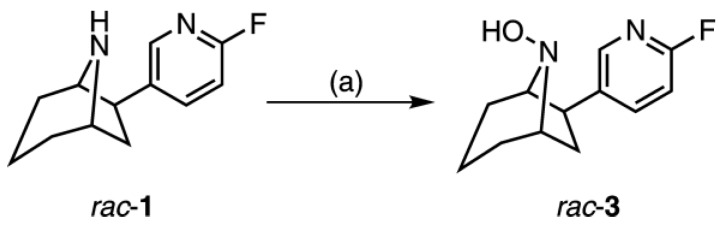
Synthesis of *rac*-8-hydroxy-flubatine (*rac*-**3**). Conditions: (**a**) HOOAc, 1 M NaHCO_3_, room temperature, six days, 9%.

*Rac*-8-Hydroxy-flubatine was synthesized starting from *rac*-**1** [2] to enable identification of the corresponding metabolite, as well as the radiometabolite by HPLC co-injection (Figure 2). After treatment with peracetic acid the desired hydroxy compound was isolated in low yield due to low conversion of the starting material. Attempts to purify *rac-***3**, e.g. by preparative HPLC, did not succeed. This can be explained by lack of stability of the *N*8-hydroxy function of the molecule, as it has been proposed in literature for metabolites that contain a secondary hydroxylamine structure [25].

### 2.3. Investigation of (+)-**1** in Pig–Identification of Phase I and Phase II Metabolites by LC-MS/MS

Since the structures of radiometabolites could not be identified in a direct manner, non-labelled (+)-**1** instead of (+)-[^18^F]**1** was investigated and administered to pigs. Plasma and urine samples were taken before, as well as 30 and 45 min, respectively, after injection. Samples were prepared by protein precipitation with cold acetonitrile, subsequent centrifugation, and solvent evaporation. In a first survey, the samples were screened for a set of possible phase I and phase II metabolites with aid of the metabolite identification software LightSight (Version 2.3.0.152038, AB SCIEX, Framingham, MA, USA). On this basis, detailed analyses were performed using multi reaction monitoring (MRM), enhanced product ion (EPI) and MS^3^ scan modes for selective detection and structure elucidation of metabolic degradation products. 

**Figure 3:**
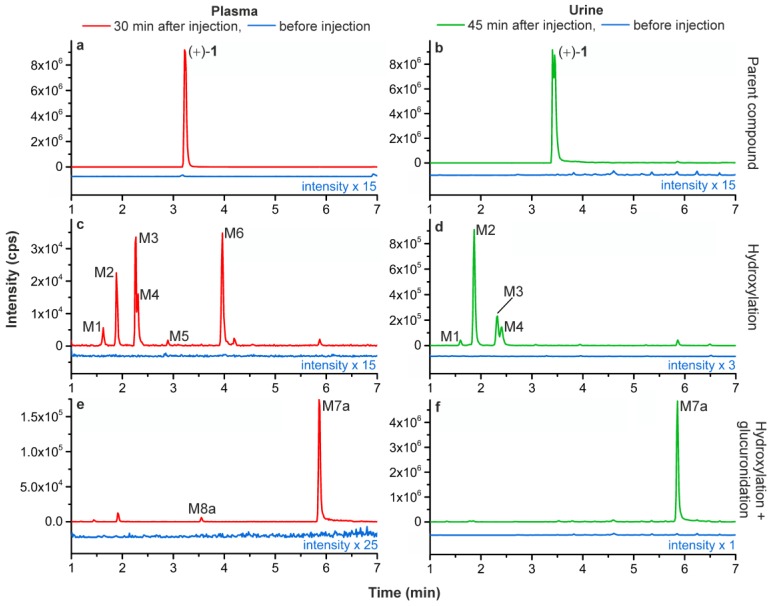
LC-MS/MS chromatograms of plasma and urine before and after injection of (+)-**1** into pigs; (**a**,**b**) detected (+)-**1** (MRM *m/z* 207.1/110.0), (**c**,**d**) detected monohydroxylated metabolites (MRM *m/z* 223.1/110.0), (**e**,**f**) detected monohydroxylated and glucuronidated metabolites (MRM *m/z* 399.2/223.1). Signals in (**c**,**d**) at 5.9 min and (**e**) at 1.9 min are supposed to result from ion-channel cross talks [26].

In plasma and urine, (+)-**1** (*m/z* 207.1 [M + H]^+^) was still detectable with high signal intensities which indicates a high metabolic stability (Figure 3a,b). However, a series of metabolites was detected, generated by cytochrome P_450_-dependent hydroxylation reactions. Appropriate MRM scans revealed that monohydroxylations took place exclusively at the azabicyclic ring system of the flubatine molecule. In brief, the chromatograms (Figure 3c,d) were recorded observing an MRM transition of *m/z* 223.1/110.0, which selectively detected a fluoro-azatropylium ion (*m/z* 110.0), that originated from an unmodified fluoropyridyl mojety after fragmentation of the parent ion at *m/z* 223.1 [M–_flubatine_ + O + H]^+^. The pattern of metabolites in pigs detected in this way was similar to those previously reported for in vitro experiments using MLM and HLM in the presence of NADPH [4]. Since, in the presented study, the same LC conditions were used as described in the mentioned publication [4], a direct comparison of data was possible. In summary, all of the hydroxylated products M1–M6 found in pigs were also formed in MLM, but only M4 and M6 in HLM. For the purpose of identification and assignment of the metabolites detected in pig enhanced product ion (EPI) spectra were recorded (Table 1). The references *rac*-**2a** and *rac*-**2b** [4], as well as the newly-synthesized *rac*-**3** (Figure 1) were analysed in an analogue manner. 

For plasma and urine samples, some of the metabolites (M3, M4) showed differing retention times. Since a mixture of both samples showed no additional peaks, this can be attributed to influences of the respective matrices. The metabolites M1 and M2 were identified as the C-3 *exo* and the C-3 *endo* alcohol, respectively, as their retention times and fragmentation patterns matched that of the references *rac*-**2a** and *rac*-**2b** (Figure 1). M3 and M4 had the same retention properties as two metabolites reported for MLM incubation [4] and were concluded to be identical to them. M4 also matches one main metabolite formed by HLM [4], additionally confirmed by similar EPI spectra. In contrast, M3 showed an expected MS/MS fragmentation but the exact pattern did not correspond to the equally eluting metabolite reported for MLM [4]. For M5 and M6 no EPI spectra could be recorded due to low concentrations. However, for M3 and M4 the characteristic fragment ion at *m/z* 188.1 [M + H-NH_3_-H_2_O]^+^ could be detected, which gives evidence for *C*-hydroxylations at the azabicyclic ring system, according to the proposed fragmentation pathway already published [4]. The same has to be assumed for M5, since its retention time is equal to a *C*-hydroxylated metabolite previously detected in considerable amounts only after MLM incubation of (−)-flubatine [4]. Metabolite M6 eluted at the same time as the synthesized reference *rac*-**3** and accordingly was identified as *N*8-hydroxylated flubatine, which was also observed after incubations with MLM or HLM in the cited study [4]. For the *C*-hydroxy isomers M3, M4, and M5, a more exact assignment of their structures was not possible, also due to limitations in syntheses of the respective references.

Furthermore, the phase II metabolite M7a was found. It was detected in both plasma and urine, when an MRM transition of *m/z* 399.2/223.1 was monitored (Figure 3e,f and Figure 5). The resulting neutral loss of *m*/*z* 176.0 (C_6_H_8_O_6_) is characteristic for glucuronides. This proved that M7a was formed by hydroxylation and glucuronidation of (+)-**1**. By contrast, a product of a sole glucuronidation of (+)-**1** could not be detected. In MS^3^ experiments for M7a, the primary fragment ion at *m/z* 223.1 [M + H-C_6_H_8_O_6_]^+^ showed a fragmentation pattern very similar to that of the 8-hydroxy-flubatine reference *rac*-**3**. In particular the occurrence of a secondary fragment ion at *m/z* 190.1 [M + H-C_6_H_8_O_6_-NH_2_OH]^+^ gave evidence for an *N*8-hydroxyl substitution present in the detected glucuronide M7a. Additionally, another glucuronide isomer (M8a) was detectable in very small amounts in plasma, but not investigated further. 

A few additional MRM measurements showed low signal intensities and gave only indications for further metabolites. For the MRM transition of *m/z* 255.1/126.0 one peak was detected at low retention time, which could be interpreted as a product of a two-fold hydroxylation at the azabicyclic ring system together with a hydroxylation or oxidation at the pyridine ring. Some data indicated cysteine conjugation of hydroxylation products, but they also were of only minor importance for the metabolic pathway.

### 2.4. In Vitro Glucuronidation of (+)-[^18^F]**1**, (+)-**1**, (−)-**1**, and rac-**3** by HLM

Since hydroxylation together with subsequent glucuronidation turned out as the significant metabolic pathway in pig, in vitro experiments were done to investigate both steps. Whilst hydroxylation of (+)-**1** and (−)-**1** has already been studied and reported [4], glucuronide conjugation was investigated for the first time. In preparation for analyses of samples from the clinical PET study radiolabeled (+)-[^18^F]**1** was investigated similarly.

In brief, carrier added (+)-[^18^F]**1** was incubated with HLM under conditions for oxidation and glucuronidation, that means in the presence of NADPH and uridine 5′-diphosphoglucuronic acid trisodium salt (UDPGA). Prepared samples were analysed by LC-MS/MS, as well as radio-HPLC. Furthermore, (−)-**1** was incubated in the same manner, whereas the *N*8-hydroxy reference *rac*-**3** was incubated without NADPH. 

**Figure 4:**
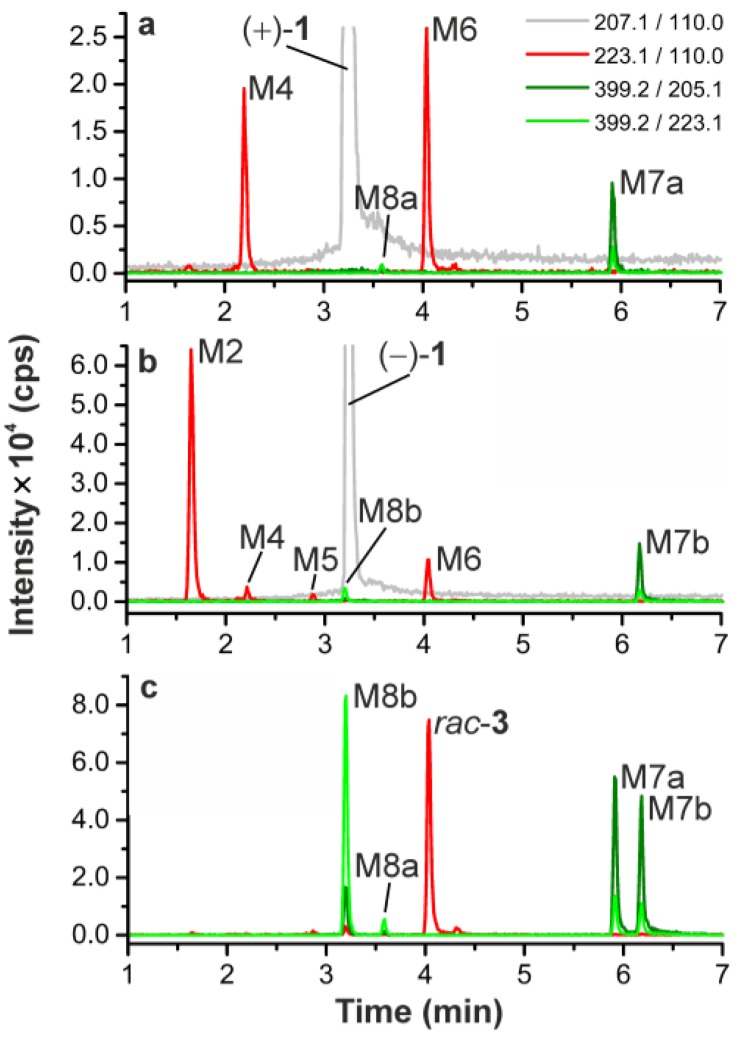
LC-MS/MS chromatograms after HLM incubation in presence of NADPH and UDPGA (37 °C, 120 min), (**a**) (+)-**1**, (**b**) (−)-**1**, (**c**) *rac*-**3** (without NADPH); Monitored MRM transitions: *m/z* 207.1/110.0 for (+)-**1** and (−)-**1**, *m/z* 223.1/110.0 for monohydroxylated metabolites (including *rac*-**3**), *m/z* 399.2/205.1, 399.1/223.1 for monohydroxylated and glucuronidated metabolites.

**Figure 5:**
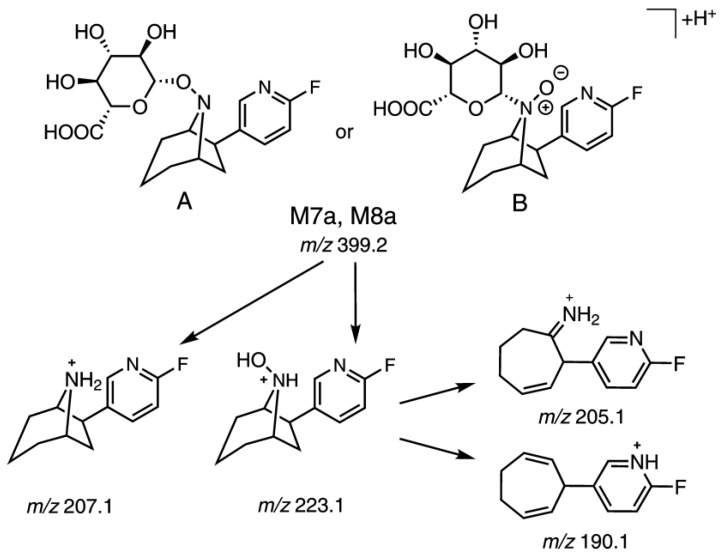
Proposed structures and fragmentation pathways shown for glucuronide conjugates M7a and M8a, drawn as N-*O*-glucuronide (**A**) and N^+^(O^−^)-glucuronide (**B**); similarly, for enantiomers M7b and M8b.

For both flubatine enantiomers (+)-**1** and (−)-**1** monohydroxylation products were observed by LC-MS/MS after HLM incubations as previously described by our group (Figure 4a,b) [4]. Originating from (+)-**1** and in accordance with results published, only two metabolites were detected, namely a *C*-hydroxylated metabolite (M4) and *N*8-hydroxylated flubatine (M6), which were also detected in pig in addition to other metabolites. Regarding the formation of glucuronides, studied for the first time, there was no glucuronidation of (+)-**1** or (−)-**1**, when each of them was incubated with UDPGA in absence of NADPH. Whereas in the presence of NADPH and UDPGA, under conditions for oxidation and glucuronidation, two products, M7a and M8a or M7b and M8b, were detected respectively by monitoring MRM transitions of *m/z* 399.2/223.1 or *m/z* 399.2/205.1 (Figure 4a,b, Table 2). As shown in Figure 4c, all of these four glucuronide isomers were formed when racemic 8-hydroxy-flubatine (*rac*-**3**) was incubated in the presence of UDPGA. This proves the *N*8-hydroxy metabolite M6, which corresponds to *rac*-**3**, to be the precursor metabolite for glucuronide conjugates. It should be noted that glucuronide M7a was formed in vitro as well as in vivo from (+)-**1**. Though having demonstrated the role of M6, further structural elucidation of the resulting glucuronide conjugates is challenging. Both the *N*8-hydroxy function and the pyridine-*N* of the molecule are possible sites for glucuronidation [27]. The finding that in the presence of UDPGA only, the *N*8-hydroxy derivative *rac*-**3** underwent glucuronidation, but in contrast not (+)-**1**, might give an indication for assignment. Due to the obvious necessity of the *N*8-hydroxy function for glucuronidations, the hypothesis can be proposed that the pyridine-*N* of the molecule remained unaffected. However, conjugation at the *N*8-hydroxy function gives the possibility of two structural isomers that can be formed: N-*O*-glucuronide and N^+^(O^−^)-glucuronide (Figure 5), which cannot be conclusively distinguished by mass spectrometry [28]. Therefore, the metabolites M7a, M7b, M8a, and M8b were tentatively identified as N-*O*-glucuronides and N^+^(O^−^)-glucuronides without an exact assignment. Remarkably, M7a and M7b eluted at later time points than the parent enantiomers, which is very untypical for glucuronides under RP-HPLC conditions, as it can be seen by comparison with retention properties of glucuronides reported in literature [29]. What kind of retention mechanism might be responsible for this phenomenon remains an open question. 

**Figure 6:**
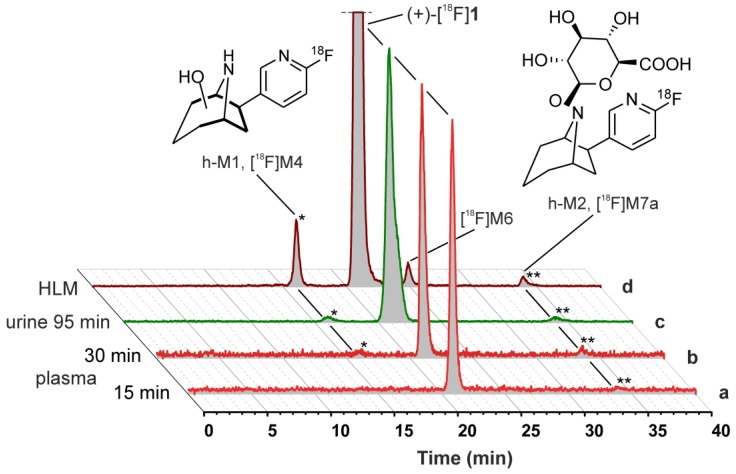
Comparison of metabolic profiles of (+)-[^18^F]**1** (in vivo vs. in vitro) and identification of radiometabolites. Representative radio-HPLC chromatograms of samples obtained from human subjects: (**a**) plasma, 15 min, (**b**) plasma, 30 min, (**c**) urine, 95 min after injection, as well as (**d**) after HLM incubation (NADPH, UDPGA, TRIS, pH 8.4, 37 °C, 120 min). Scaling was adjusted for each chromatogram.

Corresponding to the pattern of non-labelled metabolites detected by LC-MS/MS after HLM incubation of carrier added (+)-[^18^F]**1** (Figure 4a), radio-HPLC data revealed the respective in vitro radiometabolites [^18^F]M4, [^18^F]M6, and [^18^F]M7a (Figure 6). These data were used for the identification of radiometabolites formed in humans. 

### 2.5. Metabolism Studies of (+)-[^18^F]**1** in Humans

#### 2.5.1. Metabolic Stability of (+)-[^18^F]**1** and Detection of Radiometabolites

For identification of radiometabolites formed in human, arterial blood was received from two AD patients and eight healthy volunteers taken during PET measurements at 15 and 30 min after injection of of (+)-[^18^F]**1**. Urine was collected from 14 subjects uniquely after 90–128 min. After centrifugation of blood, obtained plasma was extracted with acetonitrile and the recovery of total radioactivity was 95.4 ± 2.9% (mean ± SD, *n* = 17; samples: 15 and 30 min after injection). Prepared samples of plasma and untreated urine were analysed by radio-HPLC (Figure 6).

Fractions of non-metabolised (+)-[^18^F]**1** remained very high in all samples investigated. In plasma, 15 min and 30 min after injection 97.4 ± 2.7% (mean ± SD, *n* = 10) and 95.9 ± 1.9% (*n* = 10) of (+)-[^18^F]**1** remained unchanged, respectively, while 95.1 ± 4.5% (*n* = 14) were found for urine. Due to the low number of samples from patients with AD, data could not be compared statistically with that from human controls. However, analyses by radio-HPLC revealed no obvious differences in metabolic degradation between both groups. 

One very fast eluting degradation product was detectable at very short retention times (3.5–4.0 min) only in seven of the plasma samples analysed in total and in five of the samples from urine. The signal represented, on average, 1.2% and 0.6% in plasma, 15 min and 30 min after injection, respectively, and 0.5% in urine. Due to its retention properties, it might correspond to metabolically-formed [^18^F]fluoride, but was not investigated further. Generally, detection and quantification of degradation products were limited due to low peak intensities, as shown by representative radio-HPLC chromatograms in Figure 6. However, two formed radiometabolites could be detected. Radiometabolite h-M1, which had a shorter retention time than (+)-[^18^F]**1**, represented 0.0−2.5%, 0.0−3.8%, and 0.0−4.9% in plasma, 15 and 30 min after injection, and urine, respectively. For radiometabolite h-M2, which eluted after a longer retention time, fractions of 0.0−2.6%, 0.0–4.0%, and 0.4–10.7% were determined in the same samples. 

#### 2.5.2. Identification of Radiometabolites of (+)-[^18^F]**1** formed in Humans 

Since samples obtained from human subjects were measured under the same chromatographic conditions as samples from HLM incubations of (+)-[^18^F]**1**, radio-HPLC data were used for comparison and assignment of the radiometabolites found in vivo (Figure 6). Both h-M1 and h-M2 are in good accordance with the in vitro metabolites [^18^F]M4 and [^18^F]M7a, respectively, regarding their retention times. Therefore, both radiometabolites could be identified, namely h-M1 as a product of a *C*-hydroxylation at the azabicyclic ring system and h-M2 as a glucuronide conjugate of the N8-hydroxylated (+)-[^18^F]**1** (Figure 6).

## 3. Discussion

The presented results should be seen in the context of previous metabolism studies on both enantiomers of flubatine and [^18^F]flubatine [3,4,5,6,7,8,9,10,11]. In particular, the characterisation of phase I metabolites of (+)-**1** formed by HLM and MLM, as well as the assignment of corresponding incubated with radiometabolites in mouse, published by our group [4], are highly relevant for the discussion.

As shown, monohydroxylations at the azabicyclic ring system of (+)-**1** were the major phase I metabolism pathways in pig. This is in accordance with the previously reported NADPH-dependent degradation by HLM and MLM [4], while the metabolite pattern originating from pigs was more similar to the latter. In pigs, glucuronidation of the precedingly-formed *N*8-hydroxy-flubatine (M6) revealed as very dominant and could also be demonstrated, as reported here, by incubations with HLM in presence of NADPH and UDPGA. It can be concluded that the resulting glucuronide conjugate M7a plays a major role in the urinary excretion of (+)-**1**, which, however, exhibited considerable stability. 

As described in the forementioned publication [4], after injection of (+)-[^18^F]**1** in mouse, one major radiometabolite could not be characterized but was assumed to be a phase II metabolite. Having comparable chromatographic properties it is now plausible that this radiometabolite corresponds to the glucuronide [^18^F]M7a (h-M2). The formation of [^18^F]M7a can also be concluded for one radiometabolite, that was mentioned in a previous study, that investigated (+)-[^18^F]**1** in pigs [5].

In human, radio-HPLC analyses from plasma and even urine samples revealed very high fractions of (+)-[^18^F]**1**. In comparison, with results already published for (−)-[^18^F]**1**, which showed a fraction of the unchanged tracer of ~91% [9] and ~93% [10], 30 min after injection, we found that (+)-[^18^F]**1** exhibited on average a fraction of 96% of unchanged tracer at the same time point. In vitro and in vivo studies together with LC-MS/MS and radio-HPLC served successfully as means for characterization of radiometabolites formed. Thus, despite their very low fractions, two degradation products could be detected and characterized and statements about the metabolic pathway in human could be made: In addition to its high stability, (+)-[^18^F]**1** underwent a *C*-hydroxylation at the azabicyclic ring system to form h-M1, which was equal to the in vitro formed [^18^F]M4. Hydroxylation at the bridgehead nitrogen *N*8 and subsequent conjugation with glucuronic acid resulted in the phase II radiometabolite h-M2, which was equal to the in vitro formed [^18^F]M7a. In summary, the radiometabolites detected in human were in good agreement with those formed by HLM. However, in pigs, a larger number of *C*-hydroxylation products was found.

The metabolism of the enantiomer (−)-[^18^F]**1** has already been investigated in preclinical in vivo studies [5,7,8] and in human [3,9,10], but without structural elucidation of radiometabolites. It has been reported, that in human one single metabolite was detected in low amounts by radio-HPLC but not characterized [3]. On the basis of the results presented here, it can be assumed that this radiometabolite corresponds to M7b. That means it is a product of *N*-hydroxylation of (−)-[^18^F]**1** and a subsequent glucuronidation. 

Penetration of the blood-brain barrier by radiometabolites has a high impact on the quality of brain PET measurements. The structures of the radiometabolites of (+)-[^18^F]**1,** namely h-M1 and h-M2, that were identified in the present paper, show no indications, e.g., high lipophilicity, to penetrate the human blood-brain barrier. This is in accordance with preclinical metabolism studies performed in mice, in which a low fraction of radiometabolites (< 7%) was detected in the brain, 30 min after injection [4]. 

Based on the present results and previous metabolism studies [4,5], (+)-[^18^F]**1** could be conclusively assessed regarding its metabolism. Indications for high metabolic stability, as they were obtained previously from in vitro studies [4], could be validated in vivo. In particular, in human, a fraction of 95.9 ± 1.9% (*n* = 10) of the unchanged tracer was detected in plasma, 30 min after injection. Incubations with liver microsomes served as appropriate means to generate in vitro metabolites and radiometabolites, that revealed relevant in vivo. Subsequently, in human, the two radiometabolites detected could be characterized regarding their structures. However, due to the very low extent of metabolic degradation of (+)-[^18^F]**1**, metabolism does not have to be considered in the description of the arterial input function for the kinetic modelling of PET data.

## 4. Materials and Methods

### 4.1. Chemicals and Reagents

Solvents for synthesis were purchased from Merck (Darmstadt, Germany) and Fisher Scientific (Schwerte, Germany). Chemicals were obtained from Merck, Fisher Scientific, Sigma-Aldrich (Steinheim, Germany), C. Roth (Karlsruhe, Germany) and Machery-Nagel (Düren, Germany). (+)-**1**, (−)-**1**, and *rac*-**1** were purchased from ABX (Radeberg, Germany). All chemical reagents were of highest commercially available quality and applied without further purification. The references *rac*-**2a** and *rac*-**2b** were synthesized as previously reported [4]. Acetonitrile (gradient grade) was purchased from VWR International (Darmstadt, Germany). Acetonitrile and water (both for LC-MS) were purchased from Fisher Scientific. Ammonium formate (for HPLC) was purchased from Acros Organics (Geel, Belgium). Formic acid and ammonium formate (both LC-MS), testosterone, NADPH, UDPGA, alamethicin and MgCl_2_ were purchased from Sigma-Aldrich. GIBCO human liver microsomes (HLM, pooled donors, 20 mg/mL) were purchased from Life Technologies (Darmstadt, Germany). Dulbecco’s phosphate-buffered saline (PBS) (without Ca^2+^, Mg^2+^) was purchased from Biochrom (Berlin, Germany). 

### 4.2. Radiosynthesis of (+)-[^18^F]**1**

(+)-[^18^F]**1** was synthesized under GMP conditions for human application as described for (−)-[^18^F]**1** [23].

### 4.3. Synthesis of Reference Compound rac-**3**

Reaction monitoring was performed by thin-layer chromatography (TLC) using TLC plastic sheets precoated with UV254 fluorescent indicator (Polygram SIL G/UV254, Machery-Nagel). Visualization of spots was effected by irradiation with an UV lamp (254 nm and 366 nm; Herolab, Wiesloch, Germany). ^1^H Nuclear magnetic resonance (NMR) spectra were obtained with a Bruker AV500 spectrometer (Bruker Corporation, Billerica, MA, USA). Chemical shifts are reported as δ values. Coupling constants are reported in Hertz. Electrospray ionisation mass spectra were obtained using a Surveyor MSQ Plus mass detector (Thermo Fisher Scientific, Dreieich, Germany).

#### 4.3.1. (+/−)-exo-6-(6-Fluoro-pyridin-3-yl)-8-aza-bicyclo[3.2.1]octan-8-ol ((*rac*)-8-hydroxy-flubatine, *rac*-**3**)

Racemic flubatine (*rac*-**1**) (75 mg, 0.36 mmol) was suspended in 10.7 mL 1M sodium bicarbonate. Peracetic acid (39%, 0.62 mL, 10 eq.) was added dropwise and the reaction mixture was stirred at room temperature. Further 10.7 mL 1 M sodium bicarbonate and 0.62 mL peracetic acid (39%) were added after 1, 2, and 3 days. The reaction mixture was diluted with dichloromethane after stirring for three further days. The phases were separated and the aqueous phase was extracted two times with dichloromethane. The combined organic phases were dried over sodium sulphate and the solvent was removed in vacuo. The crude product was purified by column chromatography (hexane: ethyl acetate = 1:1 + 1% triethylamine) to afford *rac*-**3** (7.5 mg, 9%) and unreacted starting material *rac*-**1** (36.2 mg, 48%). ^1^H-NMR *rac*-**3**, purity ca. 80% (CDCl_3_ , 500 MHz): δ = 8.19 (d, *J = *2.3 Hz, 1H), 8.11 (dt, *J = *8.2 Hz, 2.6 Hz, 1H), 6.83 (dd, *J = *8.6 Hz, 3.0 Hz, 1H), 3.70-3.77 (m, 1H), 3.62 (bd, *J = *1.4 Hz, 1H), 3.27 (dd, *J = *9.4 Hz, 6.9 Hz, 1H), 2.30-2.38 (m, 1H), 2.19 (dd, *J = *13.0 Hz, 9.7 Hz, 1H), 1.43-1.87 (m, 7H). MS (ESI +): *m*/*z* 223.2 (M + H)^+^. 

### 4.4. In Vivo Metabolism Study of (+)-**1** in Pigs 

The animal experiment was conducted under procedures approved by the respective State Animal Care and Use Committee and in accordance with the German Law for the Protection of Animals and the EU directive 2010/63/EU.

A female piglet (German landrace, 15 kg, eight weeks of age, obtained from Medizinisch-Experimentelles Zentrum, Universität Leipzig, Leipzig, Germany) was used. It was deprived of food, but not of water, for 24 h before delivery to the laboratory. As premedication it received an i.m. injection of 15 mg midazolam and was initially anesthetized with 1.5% isoflurane in 70% nitrous oxide and 30% oxygen. Additionally, all incision sites were infiltrated with 1% lidocaine. The anaesthesia was maintained throughout the surgical procedure with 0.8% isoflurane. After blank samples (t = 0 min) of blood and urine were taken, (+)-**1** was infused as bolus of 67 µg/kg in 50 mL saline into the left jugular vein over 6 min. After 30 and 45 min samples of blood and urine, respectively, were taken and stored on ice until further treatment. Plasma was obtained after centrifugation of blood at 4000 rpm (Megafuge 1.0R, HERAEUS) for 10 min and, using 14–20 aliquotes of 3 mL, respectively, extracted with acetonitrile (1:3 *v/v*, −20 °C). After shaking (5 min, Vortex-Genie 2, Bohemia, New York, NY, USA), cooling on ice and final shaking (3 min) the samples were centrifuged at 5000 rpm (Centrifuge 5424, Eppendorf, Hamburg, Germany) for 15 min. Supernatants of combined aliquots were concentrated at 75 °C under a flow of nitrogen using the DB-3D TECHNE Sample Concentrator (Biostep, Jahnsdorf, Germany) to obtain residual volumes of ~150 µL. After filtration (Multoclear 0.45 µm, PTFE, CS-Chromatographie Service, Langerwehe, Germany) samples were stored at 4 °C until investigated by LC-MS/MS. Urine samples were prepared in the same manner.

### 4.5. Microsomal Incubations

All incubations with (+)-**1**, (−)-**1**, (+)-[^18^F]**1**, *rac*-**2a**, *rac*-**2b**, and *rac*-**3** as substrate, had a final volume of 250 µL and were performed in TRIS (pH 8.4). In the following, final concentrations are provided in brackets. TRIS, HLM (1 mg/mL), and alamethicin (50 µg/mL, from methanolic solution) were mixed and kept on ice for 15 min. Substrate (10 µM) and MgCl_2_ (2 mM) were added and the mixture was preincubated at 37 °C for 3 min. After addition of analogously-preincubated NADPH (2 mM) and UDPGA (5 mM), incubations were proceeded by gentle shaking at 37 °C for 120 min using the BioShake iQ (QUANTIFOIL Instruments, Jena, Germany). After termination by adding 1.0 mL of cold acetonitrile (−20 °C) and vigorous mixing for 30 s, the mixtures were stored at 4 °C for 5 min. Thereafter, centrifugation at 14,000 rpm (Eppendorf Centrifuge 5424) was performed for 10 min, followed by concentration of the supernatants at 50 °C under a flow of nitrogen (DB-3D TECHNE Sample Concentrator) to provide residual volumes of 40–70 µL, which were reconditioned by adding water to obtain samples of 100 µL, which were stored at 4 °C until analysis by LC-MS/MS. For HLM incubations of carrier added (+)-[^18^F]**1**, 6.7 MBq of the synthesized tracer in 20 µL TRIS was used together with (+)-**1** (10 µM) as the substrate. Prepared samples were immediately analyzed by radio-HPLC, and by LC-MS/MS at a later time. Incubations without HLM, NADPH, UDPGA, and substrates, respectively, were performed as negative controls, as well as to provide conditions only for oxidation and not glucuronidation, and vice versa. As positive controls testosterone (for oxidation) and 4-nitrophenol (for glucuronidation) were incubated at appropriate concentrations, similarly to the protocol described above. Complete conversions of both were confirmed by RP-HPLC analyses with UV detection. 

### 4.6. Investigation of (+)-[^18^F]**1** in Humans

All investigations were conducted in the framework of an approved and registered clinical study [21]. 

After injection of 259–308 MBq (mean: 285 MBq, *n* = 14) of (+)-[^18^F]**1** into two AD patients and 12 healthy controls, respectively, 16 mL of arterial blood were taken from 10 subjects at 15 min and 30 min. The samples were collected directly into S-Monovettes 9 mL K3E (SARSTEDT, Nümbrecht, Germany) and stored on ice. From 14 subjects, after 90–128 min, circa 8 mL of urine were collected uniquely and stored on ice. Plasma was obtained by centrifugation of blood samples at 7000 rpm (UNIVERSAL 320 R, Hettich, Germany) for 7 min and extracted with acetonitrile (1:4 *v/v*, 10–15 aliquotes of 400 µL each). After addition of the cold extraction solvent (−35 °C) and shaking for 3 min, samples were cooled at 4 °C, shaken for a further 3 min, and centrifuged at 7000 rpm (Eppendorf Centrifuge 5424) for 5 min. The concentration of the supernatants at 50 °C under a flow of nitrogen (Sample Concentrator DB-3D TECHNE) provided residual volumes of 40–70 µL, which were reconditioned by adding water to obtain samples of 100 µL, which were immediately analyzed by radio-HPLC. Urine samples were analyzed without any pretreatment. 

### 4.7. LC-MS/MS Analyses

Analyses were performed as previously described [4] on an Agilent 1260 Infinity Quarternary LC system (Agilent Technologies, Böblingen, Germany) coupled with a QTRAP 5500 hybrid linear ion-trap triple quadrupole mass spectrometer (AB SCIEX, Concord, ON, Canada). Data were acquired and processed using Analyst software (Version 1.6.1, AB SCIEX) and for further data processing Origin Pro 8.5.0G (OriginLab, Northampton, MA, USA) was used. For chromatographic separations a Poroshell 120 EC-C18-column, 50 mm × 4.6 mm, 2.7 μm (Agilent Technologies) was used. The solvent system consisted of eluent A: aq. ammonium formate, 2.5 mM, pH 3 and eluent B: water/acetonitrile, 20:80 (*v*/*v*), containing ammonium formate, 2.5 mM, pH 3. Gradient elution (% acetonitrile) at 25 °C and a flow rate of 1.0 mL/min: 0–9.0 min, 5–37%; 9.0–9.1 min, 37–80%; 9.1–11.0 min 80%; 11.0–11.1 min, 80–5%; 11.1–14.0 min, 5%. The mass spectrometer was operated in positive electrospray ionization mode and the following parameters were: curtain gas (CUR) 40, collision gas (CAD) high, ion spray voltage (IS) 5500, temperature (TEM) 650, ion source gas 1 (GS1) 60, and ion source gas 2 (GS2) 60. For the multiple reaction monitoring (MRM) scan type: different MRM transitions, scan time. 40 ms; declustering potential (DP), 126; entrance potential (EP), 10; collision energy (CE), 37; collision cell exit potential (CXP), 14. For the enhanced product ion (EPI) scan type: product of *m*/*z*, 223.1; scan rate, 10,000 Da/s; dynamic fill time; DP, 110; EP, 10; CE, 33; collision energy spread (CES), 0. For the EPI scan type: product of *m*/*z*, 399.2; scan rate, 10,000 Da/s; dynamic fill time; DP, 110; EP, 10; CE, 50; CES, 0. For the MS^3^ scan type the excitation energy (AF2) was optimized prior to data acquisition.

### 4.8. Radio-HPLC

Analyses were performed on a JASCO LC-2000 system (JASCO Labor- und Datentechnik, Gross-Umstadt, Germany) including a UV-2070 UV–VIS detector (monitoring at 254 nm) online with a GABI Star radioactivity flow detector (raytest Isotopenmessgeräte, Straubenhardt, Germany) with a NaI detector (2 × 2″ pinhole, 16 mm × 30 mm). Chromatographic separations were achieved using a Multospher 120 RP 18 AQ-5μ-column, 250 mm × 4.6 mm, 5 μm, including precolumn, 10 mm × 4 mm (CS-Chromatographie Service). The solvent system consisted of eluent A: aq. ammonium formate, 25 mM, pH 3/acetonitrile, 95:5 (*v*/*v*) and eluent B: aq. ammonium formate, 25 mM, pH 3/acetonitrile, 20:80 (*v*/*v*). Gradient elution (% acetonitrile) at a flow rate of 1.0 mL/min: 0–5 min, 5%; 5–40 min, 5–30%; 40–41 min, 30–80%; 41–51 min, 80%; 51–52 min, 80–5%; 52–62 min, 5%.

## 5. Conclusions

By metabolism studies in pigs and incubations with liver microsomes, supported by LC-MS/MS and radio-HPLC, radiometabolites of (+)-[^18^F]**1** formed in humans could be identified: one *C*-hydroxylation product (h-M1) and one product of *N*-hydroxylation, and subsequent glucuronide conjugation (h-M2). Due to the very low occurrence of these metabolites, (+)-[^18^F]**1** exhibits a tremendously high metabolic stability in human. (+)-[^18^F]**1** is considered as a very appropriate tracer for the molecular imaging of α_4_β_2_ nAChRs by PET.

## Figures and Tables

**Table 1:** Metabolites found in pig plasma and urine after injection of (+)-**1**.

Metabolite	t_R_ (min) ^a^		MRM Transition	EPI Fragmentation ^b^ (% Intensity in Brackets)	Identification
	Plasma	Urine			
**M1**	1.62	1.60	223.1/110.0	81.9 (100), 205.0 (71), 223.0 (32), 163.1 (26), 162.1 (23), 110.0 (17), 136.0 (15), 131.0 (15), 103.9 (14), 188.1 (9)	Hydroxylation
**M2**	1.89	1.87	223.1/110.0	223.1 (100), 136.0 (40), 110.0 (39), 162.1 (37), 180.1 (36), 188.0 (28), 179.1 (25), 114.0 (13), 124.0 (13), 206.0 (11), 160.0 (9), 138.0 (8), 142.0 (4), 205.1 (4)	
**M3**	2.26	2.32	223.1/110.0	223.1 (100), 150.0 (63), 164.1 (44), 110.0 (42), 188.1 (41), 82.0 (39), 135.1 (31), 206.1 (18), 176.1 (16), 120.1 (14), 162.1 (12), 124.0 (12), 205.1 (12), 134.0 (12), 136.1 (12), 163.1 (10)	
**M4**	2.31	2.40	223.1/110.0	81.9 (100), 205.1 (60), 188.0 (39), 177.1 (31), 223.1 (23), 110.0 (22), 176.1 (9), 160.0 (7)	
**M5**	2.89	*-* ^c^	223.1/110.0	no EPI spectrum due to low intensity	
**M6**	3.96	*-* ^c^	223.1/110.0	no EPI spectrum due to low intensity	
**M7a**	5.87	5.86	399.2/223.1	205.2 (100), 223.2 (44), 136.1 (34), 203.2 (26), 110.0 (13), 162.1 (11), 177.2 (9), 83.0 (9), 207.2 (8), 189.1 (6), 109.1 (6), 190.2 (6)	Hydroxylation + glucuronidation

^a^ Retention time (time/min); ^b^ parameters for data acquisition described in Section 4.7.; ^c^ not detected in urine.

**Table 2:** Glucuronide conjugates formed by incubation with HLM.

Metabolite ^a^	t_R_ (min) ^b^	Substrate	MRM Transitions	EPI Fragmentation ^c^ (% Intensity in Brackets)	Identification
**M7b**	6.18	(−)-**1** or *rac*-**3**	399.2/223.1 399.2/205.1	205.1241 (100), 223.1 (60), 136.0 (37), 203.1 (20), 110.0 (18), 177.1 (16), 83.0 (11), 207.1 (11), 162.0 (11), 82.0 (10), 116.0 (8), 163.1 (8), 190.1 (7), 124.0 (7)	Hydroxylation + Glucuronidation ^d^
**M8a**	3.60	(+)-**1** or *rac*-**3**	399.2/223.1 399.2/205.1	223.1 (100), 205.1 (21), 124.1 (6), 110.2 (6), 136.0 (6), 84.9 (4), 113.0 (4)	
**M8b**	3.22	(−)-**1** or *rac*-**3**	399.2/223.1 399.2/205.1	223.1 (100), 205.1 (27), 136.1 (16), 84.9 (9), 110.0 (8), 150.0 (6), 203.2 (6), 82.9 (6), 67.9 (4), 190.1 (4), 113.0 (3)	

^a^ data for M7a, formed from (+)-**1** or *rac*-**3**, correspond to those stated in Table 1; ^b^ Retention time (time/min); ^c^ parameters for data acquisition described in Section 4.7.; ^d^ for *rac*-**3** glucuronidation only.
